# Ebosin Attenuates the Inflammatory Responses Induced by TNF-*α* through Inhibiting NF-*κ*B and MAPK Pathways in Rat Fibroblast-Like Synoviocytes

**DOI:** 10.1155/2022/9166370

**Published:** 2022-03-17

**Authors:** Yang Zhang, Lifei Wang, Liping Bai, Rong Jiang, Jianbo Wu, Yuan Li

**Affiliations:** ^1^NHC Key Laboratory of Biotechnology of Antibiotics, CAMS Key Laboratory of Synthetic Biology for Drug Innovation, Institute of Medicinal Biotechnology, Chinese Academy of Medical Sciences & Peking Union Medical College, No. 1 Tiantan Xili, 100050 Beijing, China; ^2^Beijing Institute of Hepatology, Beijing Youan Hospital, Capital Medical University, Beijing 100069, China

## Abstract

Tumor necrosis factor-*α* (TNF-*α*) lies at the apex of signal transduction cascades that results in induced destruction of joints in rheumatoid arthritis. It is therefore of great medicinal interest to modulate the cellular responses to TNF-*α*. Ebosin, a novel exopolysaccharide derived from *Streptomyces* sp, has been demonstrated to have remarkable therapeutic actions on collagen-induced arthritis in rats, while it also suppressed the production of IL-1*β*, TNF-*α*, and IL-6 at both mRNA and protein levels in cultured fibroblast-like synoviocytes. In order to further understand the potential mechanisms involved in the anti-inflammatory effects of ebosin at molecular level, we investigated the impact of it on the activation of MAPK and NF-*κ*B pathways following TNF-*α* induced in fibroblast-like synoviocytes (FLS). The results showed that the phosphorylation levels of TNF-*α*-induced p38, JNK1, JNK2, IKK*α*, IKK*β*, and I*κ*B, as well as NF-*κ*B nuclear translocation, were reduced significantly in FLS cells in response to ebosin. Furthermore, we proved that ebosin decreased the level of NF-*κ*B in the nucleus and blocked the DNA-binding ability of NF-*κ*B using electrophoresis mobility gel shift assay. Besides, low levels of matrix metalloproteinases (MMP-1 and MMP-3) and chemokines (interleukin-8 and RANTES) were found in TNF-*α*-stimulated fibroblast-like synoviocytes treated with ebosin. These results indicate that ebosin can suppress a range of activities in both MAPK and NF-*κ*B pathways induced by TNF-*α* in rat fibroblast-like synoviocytes, which provides a rationale for examining the use of ebosin as a potential therapeutic candidate for rheumatic arthritis.

## 1. Introduction

Rheumatoid arthritis (RA) is a chronic destructive disease of the joints and cartilage. Proinflammatory cytokines such as tumor necrosis factor (TNF) produced by fibroblast-like synoviocytes (FLS) and inflammatory cells, which are expressed at high levels in rheumatoid joint tissue, where they contribute significantly to inflammation and articular destruction. Dysfunction of TNF-*α* is involved in the pathological process of different types of diseases including RA [1, 2], which was the first proinflammatory cytokine fully identified as a therapeutic target for RA [3]. TNF-targeted therapy has convincingly demonstrated significant benefit for the majority of RA patients treated. Using recombinant proteins including infliximab, adalimumab, and golimumab [4] to block TNF has emerged in recent years.

TNF-*α* elicits its biological activities through binding to two types of cell surface receptors TNF-R1 and TNF-R2. In most of the cells, TNF-R1 was considered to be a key mediator of TNF-*α* signal [5]. NF-*κ*B is an important downstream target of TNF-*α* signaling, and it has been identified to be involved in inflammatory responses [6]. TNF-*α* triggers NF-*κ*B activation by a variety of signaling molecules, including TRAF2 (TNF receptor-associated factor 2), RIP (receptor-interacting protein), and the IKK (I*κ*B kinase) complex. Another signal pathway activated by TNF-*α* is mitogen-activated protein kinase pathways (including p38, JNK, and ERK1/2), which has been strongly associated with many of the processes that mediate the pathological features of RA [7]. The p38 MAPK and JNK (Jun N-terminal kinase) pathway inhibitors attract more attention since they can reduce both the synthesis of proinflammatory cytokine and their intracellular signaling [8]. In addition, TNF-*α* also induces extracellular matrix (ECM) remodeling by regulating the expression of MMPs involved in joint damage in RA, as well as expression of multiple chemokines including IL-8, MCP-1, CCL3, CCL4, and RANTES, which contribute to chronic inflammatory [9–16]. Currently, anti-inflammatory therapy by inhibiting MAPK and NF-*κ*B pathways has been well recognized [17].

Ebosin, a novel exopolysaccharide (EPS) extracted from *Streptomyces* sp.139 of soil samples in China, remarkably inhibits the development of CIA (collagen-induced arthritis) in rats [18], which consisted of rhamnose, fucose, arabinose, mannose, xylose, glucose, galactose, and galacturonic acid [19]. This anti-inflammatory effect of ebosin on CIA has been identified to be related to attenuating the production of IL-1*β* (interleukin-1*β*), IL-6 (interleukin-6), and TNF-*α* at the transcriptional and posttranscriptional levels [18].

Although anti-TNF agents have been shown to improve the outcome of the management of RA, a proportion of patients does not respond well to anti-TNF therapy and has increased the risk of adverse events such as infections [20–24]. Unlike antibody-based agents, ebosin is the first EPS produced by the *Streptomyces* genus with a novel structure [19]. In a previous study, we found that the inhibitory effect of ebosin on TNF-*α* secretion was stronger than other cytokines as IL-1*β* and IL-6 in the CIA rat model and FLS cells, implicating that ebosin may improve rheumatoid arthritis symptoms through the TNF-*α* signaling pathway [18]. However, ebosin may have a wide application prospect due to its efficacy and high safety which have been proven by a long-term toxicity study (unpublished data). Based on the above, the purpose of this study was to investigate the impact of ebosin on TNF-*α*-mediated MAPK and NF-*κ*B pathways, as well as on levels of MMPs and chemokines in rat fibroblast-like synoviocytes (FLS), which will help us to better understand the mechanism of action of ebosin.

## 2. Materials and Methods

### 2.1. Isolation of FLSs

Male Wistar rats (Certificate No.: SCXK 2005-0013) were obtained from the Institute of Experimental Animals, Chinese Academy of Medical Sciences, Beijing [18]. All rats were handled humanely and procedures under standard laboratory conditions with the approval of the Institute of Experimental Animals and Use Committee of the Chinese Academy of Medical Sciences. Chicken type II collagen (CII, Sigma)-induced arthritis (CIA) model and isolation of FLS were performed following the protocol described by Zhang et al. [18, 25]. In brief, Synovial tissues were isolated from the knee joint of CIA rats, which were sacrificed on day 30 after immunization, and then digested by 0.4% type II collagenase (Gibco). Isolated FLS were grown in DMEM high-glucose medium supplemented with 10% fetal bovine serum, 100 units/ml penicillin, and 100 *μ*g/ml streptomycin and cultured at 37°C, 5% CO_2_.

### 2.2. Purification of Ebosin

Ebosin-producing *Streptomyces.*139 was found from a soil sample in China and deposited in the China General Microbiology Culture Collection Center (No. 0405). Ebosin was purified from the supernatant of fermentation culture of *Streptomyces* sp.139 according to the protocol as described before [19].

### 2.3. Cytoplasmic and Nuclear Protein Extraction

FLS were cultivated in 6-well plates (at 1 × 10^6^/ml) at 37°C for 24 h. Ebosin (80, 16, or 3.2 *μ*g/ml) was diluted in DMEM and added into FLS for 3 h with TNF-*α* (10 ng/ml). Cells were collected in 0.25% trypsin-EDTA (HyClone) and harvested by centrifugation at 500 g for 5 min. Cytoplasmic and nuclear were extracted using NE-PER Nuclear and Cytoplasmic Extraction Reagents following the manufacturer's protocol [25]. The cytoplasmic and nuclear extracts were stored at -80°C. Determination of protein concentration was performed by the Bradford method [26].

### 2.4. Western Blot Analysis

The effect of Ebosin on MAPK and NF-*κ*B signaling pathways was determined by western blot assay as described previously [25]. The expression level of protein was detected with antibodies (Cell Signaling Technology) against phosphorylated or nonphosphorylated p38, JNK1, JNK2, ERK, IKK*α*, IKK*β*, I*κ*B, and NF-*κ*B p65, respectively. The relative densities for the protein bands were quantitated using ImageQuant 300 (GE Healthcare) with Image J software.

### 2.5. Enzyme-Linked Immunosorbent Assays (ELISAs)

ELISA was used to evaluate the levels of IL-8, RANTES, MMP-1, and MMP-3 in cell culture medium. FLS cells were plated in 24-well plates at 1 × 10^6^/ml and cultivated at 37°C for 24 h and then received proper ebosin treatments (80, 16, and 3.2 *μ*g/ml) for 12 h. Cells were further induced by adding 10 ng/ml TNF-*α* for 24 h before supernatants were collected. The level of MMP-1, MMP-3, RANTES, and IL-8 in the medium was detected using colorimetric ELISA kits (USCN Life Science Inc. and Boster, Elab).

### 2.6. Electrophoretic Mobility Shift Analysis (EMSAs)

EMSA was performed to detect the effect of ebosin on the ability of NF-*κ*B binding to DNA in nuclei using the LightShift Chemiluminescent EMSA Kit (Thermo) following the instructions of the manufacturer [25]. Extraction of nuclear protein was performed as previously described. Oligonucleotides labeling with biotin (5′-AGTTGAGGGGACTTTCCCAGGC-3′; 3′-TCAACTCCCCTGAAAGGGTCCG-5′), which contains the *κ*-chain binding site (*κ*B, 5′-GGGACTTTCC-3′), was synthesized from Beyotime Biotechnology Company. Unlabeled oligonucleotide and a 50-fold excess of cold *κ*B oligonucleotide were used as a control to confirm specific binding.

### 2.7. Immunofluorescence Analysis

FLS cells were cultivated in 96-well plates (at 1 × 10^4^/ml) at 37°C for 12 h. Ebosin (80 *μ*g/ml) was added in each well followed by cultivation at 37°C for 12 h and then treated cell with TNF-*α* (10 ng/ml) for an additional 3 h. The cells were fixed in 5% paraformaldehyde (PFA)/PBS for 10 min at room temperature, permeabilized with PBS/0.1% Triton-X100 for 15 min, and blocked in PBS with 5% bovine serum albumin for 1 h. The cells were incubated with rabbit anti-NF-*κ*B p65 (Cell Signaling Technology) and Alexa Fluor 488 donkey anti-rabbit IgG (Molecular Probes). The fluorescent signals were detected by fluorescence microscopy (Olympus IX71, Japan).

### 2.8. Statistical Analysis

Data are presented as mean ± SD values. Evaluation of significance of differences between sample groups was performed by GraphPad Prism software (Version 6.0) using Student's *t*-test. All *P* value < 0.05 was considered significant (∗).

## 3. Results

### 3.1. Ebosin Downregulates TNF-*α*-Induced p38 MAPK Activation

**Figure 1:**
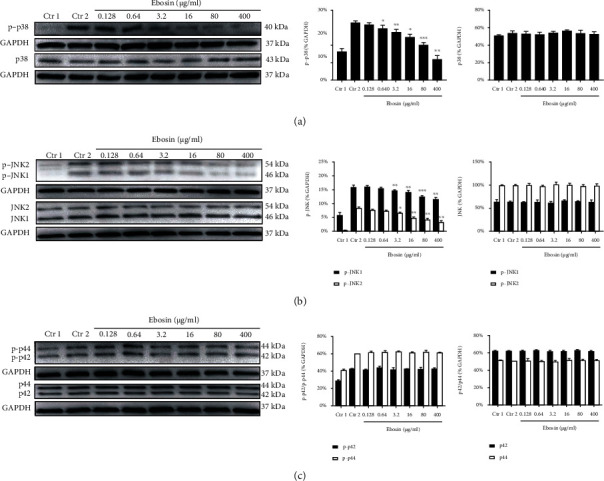
Ebosin inhibits the MAPK signaling pathway mediated by TNF-*α* in FLS. (a) Effect of ebosin on the phosphorylated and nonphosphorylated p38 MAPK separately. FLS cells (1 × 10^5^/ml) were incubated in the presence or absence of ebosin for 12 h then with TNF-*α* (10 ng/ml) 15 min. The phosphorylated and nonphosphorylated p38 MAPK were analyzed by western blot. (b) Effect of ebosin on the phosphorylated and nonphosphorylated JNK1 and JNK2 MAPK, respectively. The experimental protocol is the same as p38. (c) Effect of ebosin on phosphorylated and nonphosphorylated p42/44 MAPK (ERK1/2). The protocol is also the same as before. All of the data are expressed as the means ± SD from at least 3 independent experiments. *P* < 0.001, *P* < 0.01, and *P* < 0.05 compared to the control 2 (FLS incubated with TNF-*α*).

Phosphorylation of p38 MAPK is involved in the activation of proinflammatory cytokines including TNF-*α* [27]. In order to investigate the effect of ebosin on TNF-*α*-induced p38 MAPK activation, FLS cells were treated with ebosin at a concentration of 0.128, 0.64, 3.2, 16, 80, and 400 *μ*g/ml for 12 h. Phosphorylated active forms of p38 MAPK were detected by western blot in FLS cells following TNF-*α* stimulation. As shown in Figure 1(a), ebosin markedly reduced the level of phosphorylation of p38 MAPK by 63.67% (*P* < 0.01), 38.94% (*P* < 0.001), 25.21% (*P* < 0.05), 16.51% (*P* < 0.01), 10.16% (*P* < 0.05), and 3.62%, respectively, in a dose-dependent manner, while the levels of nonphosphorylated p38 in FLS did not change (Figure 1(a)), indicating that ebosin can downregulate p38 MAPK activation induced by TNF-*α*.

### 3.2. Ebosin Reduces the Production of Phosphorylated JNK1 and JNK2 MAPK

JNK is one major group of MAPK cascades, which are activated by TNFR superfamily members [27]. To understand the effect of ebosin on TNF-*α*-induced expression of JNKs in FLS, the supernatant of lysed cells was assessed by western blot after treating cells with TNF-*α* in the presence of ebosin. The results showed that ebosin in the range of 0.128~400 *μ*g/ml significantly reduced the expression levels of phosphorylated JNK1 by 50.32% (*P* < 0.01), 34.84% (*P* < 0.001), 27.60% (*P* < 0.01), 13.51% (*P* < 0.01), 8.95%, and 1.19%, respectively (Figure 1(b)), while the phosphorylated JNK2 levels decreased by 59.09% (*P* < 0.01), 50.07% (*P* < 0.01), 43.00% (*P* < 0.01), 21.96% (*P* < 0.05), 13.29%, and 8.30%, respectively (Figure 1(b)), but does not affect nonphosphorylated forms of JNK1 and JNK2 (Figure 1(b)) at the same dosages.

### 3.3. The Expression Levels of p42/44 MAPK (ERK1/2) Are Not Influenced by Ebosin

The activation of p42/44 MAPK is associated with inflammatory response, synovial proliferation, and angiogenesis in RA [27]. To valuate effect of ebosin on TNF-*α*-induced expression of p42/44 MAPK in FLS, we treated FLS cells with TNF-*α* in the presence of ebosin, and then, the phosphorylated (or nonphosphorylated) p42/44 protein level in the supernatant of lysed cells was determined using western blot. The results found that ebosin did not exert any action on both phosphorylated and nonphosphorylated forms of p42/44 MAPK (Figure 1(c)).

### 3.4. Effect of Ebosin on Expression of IKK*α* and IKK*β*

**Figure 2:**
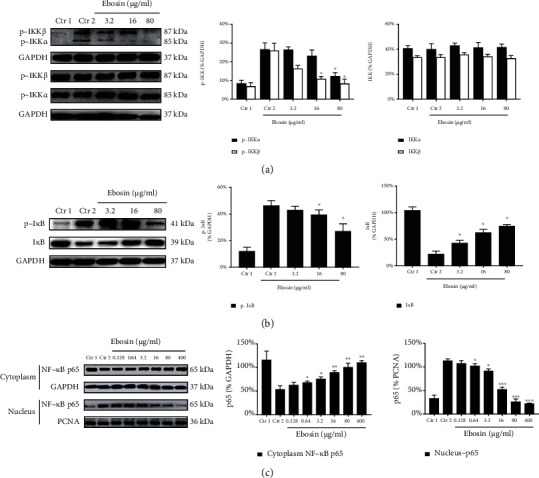
The influences of ebosin on the NF-*κ*B signaling pathway mediated by TNF-*α* in FLS. (a) Effects of ebosin on the expression of phosphorylated and nonphosphorylated IKK*α* and IKK*β*. FLS cells (1 × 10^5^/ml) were incubated in the presence or absence of ebosin for 12 h then with TNF-*α* (10 ng/ml) 3 h. The production of phosphorylated and nonphosphorylated IKK*α* and IKK*β* was analyzed with western blot. (b) Effects of ebosin on production of phosphorylated I*κ*Ba and nonphosphorylated I*κ*Ba. The expression levels of phosphorylated and nonphosphorylated I*κ*Ba were identified with the same protocols as before. (c) Effect of ebosin on nuclear and cytoplasm NF-*κ*B of FLS. The production of nuclear and cytoplasm NF-*κ*B was determined by western blot also. All of the data are expressed as the means ± SD from at least 3 independent experiments. *P* < 0.001, *P* < 0.01, and *P* < 0.05 compared to the control 2 (FLS incubated with TNF-*α*) individually for NF-*κ*B, IKK*α*, IKK*β*, and I*κ*Ba.

The IKK complex has consisted of two catalytic subunits, IKK*α* and IKK*β*, and a noncatalytic subunit IKK*γ*. Activation of IKK complex by TNF-*α* stimulation requires the phosphorylation of IKK*α*/*β* in its activation loop and polyubiquitination of IKK*γ* [28]. To expand these studies, we have analyzed the impact of ebosin on the phosphorylation level of IKK*α* and IKK*β* in response to TNF-*α*. Western blot results showed that 80, 16, and 3.2 *μ*g/ml ebosin decreased the protein levels of phosphorylated IKK*α* by 53.85% (*P* < 0.01), 19.90% (*P* < 0.05), and 0.85% separately and IKK*β* by 71.56% (*P* < 0.01), 58.11% (*P* < 0.05), and 40.28% (*P* < 0.05), respectively (Figure 2(a)), but not nonphosphorylated IKK*α* and IKK*β* (Figure 2(a)).

### 3.5. Ebosin Suppresses the Production of I*κ*B*α*

I*κ*Bs is an inhibitory factor of NF-*κ*B, and ubiquitination of I*κ*Bs leads to release NF-*κ*B entering the nucleus and activating transcription of appropriate gene targets [28]. Using western blot, we measured the expression level of TNF-*α*-induced I*κ*B*α* with ebosin in FLS cells. As shown in Figure 2(b), ebosin reduced the expression levels of phosphorylated I*κ*B*α* by 35.27% (*P* < 0.05), 14.99% (*P* < 0.05), and 6.93%, respectively, at dosages 80, 16, and 3.2 *μ*g/ml, but the levels of nonphosphorylated I*κ*B*α* were enhanced by 55.12% (*P* < 0.05), 45.08% (*P* < 0.05), and 32.78% (*P* < 0.05), respectively, at the same dosages (Figure 2(b)).

### 3.6. Ebosin Attenuates NF-*κ*B DNA-Binding Activity

**Figure 3:**
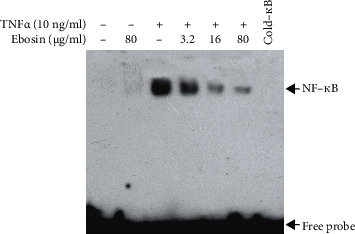
Ebosin inhibits NF-*κ*B DNA binding activity in FLS mediated by TNF-*α*. The DNA binding activity in the nuclear extracts of FLS mediated by TNF-*α* was assessed in an electrophoretic mobility shift assay (EMSA) by a specific probe, an oligonucleotide labeled with biotin. A LightShift Chemiluminescent EMSA Kit was used following the instructions of the manufacturer. Specific binding was controlled by competition with a 50-fold excess of cold *κ*B oligonucleotide.

Once released from its inactive form complexed with I*κ*Bs, NF-*κ*B is presumably translocated to the nucleus and interacted with specific DNA-binding sequences to regulate gene transcription [29]. To confirm if ebosin can affect the NF-*κ*B's activity through interfering with its specific DNA, an electrophoretic mobility shift analysis (EMSA) was performed in FLS cells stimulated by TNF-*α* and then incubation with varying concentrations of ebosin. Results in Figure 3 showed that ebosin significantly blocked NF-*κ*B-DNA binding in a dose-dependent manner.

### 3.7. Ebosin Inhibit NF-*κ*B Nuclear Translocation

**Figure 4:**
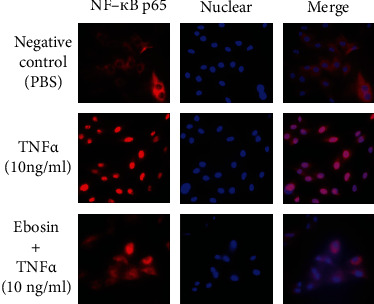
The effect of ebosin on nuclear translocation of NF-*κ*B mediated by TNF-*α* in FLS. Indirect immunofluorescence with the specific anti-NF-*κ*B p65 antibody was performed. Using a fluorescence microscope, the cells were counterstained by Hoechst 33528 for nuclear staining.

For understanding the effect of ebosin on the activity of NF-*κ*B, we detected the NF-*κ*B protein level in the cytoplasm and nucleus using the NF-*κ*B p65 antibody. Western blot analysis showed (Figure 2(c)) that ebosin markedly enhanced the protein level of p65 in the cytoplasm induced with TNF-*α* by 51.72% (*P* < 0.01), 46.84% (*P* < 0.05), 40.33% (*P* < 0.05), 30.34% (*P* < 0.05), 23.12% (*P* < 0.05), and 14.34%, respectively, at dosages 400, 80, 16, 3.2, 0.64, and 0.128 *μ*g/ml. However, it decreased the expression of NF-*κ*B in the nucleus of cells by 80.11% (*P* < 0.001), 77.02% (*P* < 0.001), 53.77% (*P* < 0.001), 19.25% (*P* < 0.05), 10.07% (*P* < 0.05), and 5.25%, respectively, at same dosages (Figure 2(c)). For further understanding the nuclear events that govern NF-*κ*B function by ebosin, immunofluorescence was performed to analyze the translocation of activated NF-*κ*B in the nucleus (Figure 4) The cells were treated with ebosin at 80 *μ*g/ml after TNF-*α* inducing and then incubated with rabbit NF-*κ*B p65 antibody. From these results, we observed that the process of NF-*κ*B from the cytoplasm into the nucleus was significantly suppressed in FLS by ebosin (Figure 4).

### 3.8. Ebosin Reduced the Secretion of MMP1 and MMP3

**Figure 5:**
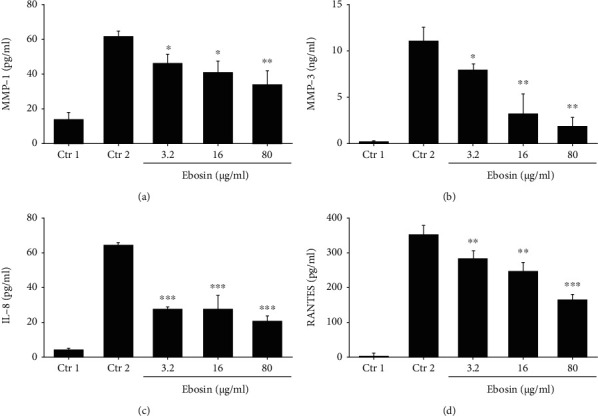
Ebosin suppresses production of MMPs and chemokines mediated by TNF-*α* in FLS. (a, b) Inhibition of ebosin on production of MMP1 and MMP3. FLS cells (1 × 10^6^/ml) were incubated in the presence or absence of ebosin for 12 h then with TNF-*α* (10 ng/ml) for 24 h. The expression levels of MMP1 and MMP3 were analyzed by ELISA kits individually. (c, d) Influences of ebosin on production of chemokines including RANTES and IL-8. The levels of RANTES and IL-8 were determined also by ELISA kits separately. *P* < 0.001, *P* < 0.01, and *P* < 0.05 compared to the control 2 (FLS incubated with TNF-*α*).

The increased level of MMP expression has been associated with destroy collagenous components of cartage in RA [30]. In order to study the therapeutic effects of ebosin, we measured the influence of ebosin on MMP-1 and MMP-3 secretion in TNF-*α*-induced FLS cells by ELISA. As shown in Figure 5(a), the concentration of MMP-1 in cell-cultured supernatant decreased by 44.16% (*P* < 0.01), 33.63% (*P* < 0.05), and 26.94% (*P* < 0.05), respectively, at dosages of 80, 16, and 3.2 *μ*g/ml of ebosin, and meanwhile, the productions of MMP-3 in FLS were suppressed by 83.01% (*P* < 0.01), 70.81% (*P* < 0.01), and 32.63% (*P* < 0.05), respectively (Figure 5(b)).

### 3.9. Effect of Ebosin on Secretion of Chemokines

Clinical studies have demonstrated chemokines, produced by FLS cells, promoting inflammation and cartilage destruction in response to TNF-*α* [31]. In this study, we detect the concentration of TNF-*α*-induced release of chemokines comprising RANTES and IL-8 in FLS treated with ebosin by ELISA. The results showed that ebosin at dosages 80, 16, and 3.2 *μ*g/ml decreased the production of IL-8 by 68.08% (*P* < 0.001), 57.52% (*P* < 0.001), and 57.28% (*P* < 0.001), respectively (Figure 5(c)), and at the meantime, diminished the expression levels of RANTES by 53.08% (*P* < 0.001), 29.94% (*P* < 0.01), and 19.93% (*P* < 0.01) separately (Figure 5(d)).

## 4. Discussion

**Figure 6:**
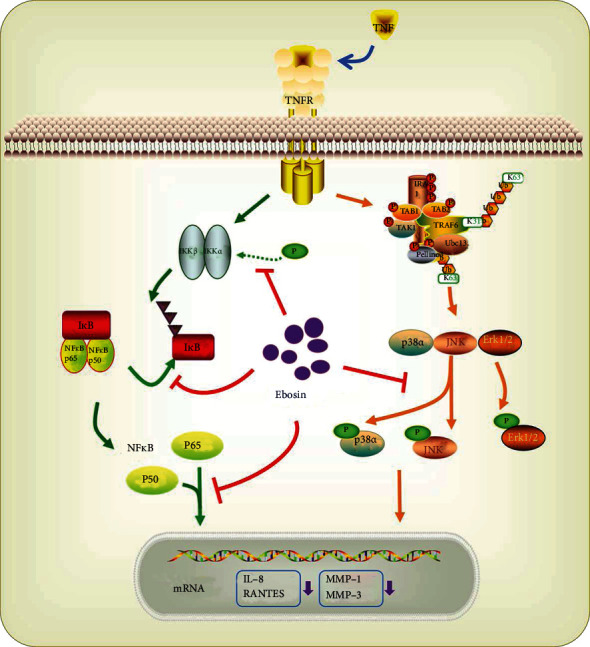
Schematic representation of TNF-*α*-induced activation of MAPK and NF-*κ*B signaling pathways modulated by ebosin. The biological effects of TNF-*α* were attenuated by ebosin on two different pathways. First, phosphorylation of p38 and JNK MAPK was significantly inhibited by ebosin with a dose-dependent manner, which may contribute to reduce the nuclear translocation of p38 and JNK. Secondly, it has been shown ebosin can significantly decrease the phosphorylation level of IKK*α* and IKK*β*. And then, the increased level of I*κ*B with a concomitant decrease in the level of p-I*κ*B resulted in cytoplasmic retention of NF-*κ*B p65 and depression of the DNA binding ability of NF-*κ*B in the nucleus. Finally, the expression of MMP-1, MMP-3, interleukin-8, and RANTES which induced by TNF-*α* was significantly reduced responding to ebosin treatment in FLS cells.

In the current study, we demonstrated that ebosin affects the TNF-*α*-induced inflammatory responses in FLS largely due to its intervention in TNF-*α*-induced MAPKs and NF-*κ*B pathways (Figure 6).

Inflammation, a representative innate immune response and the cause of numerous diseases including cancer and arthritis [32], is characterized by the involvement of a common set of genes and endogenous mediators including growth factors, inflammatory cytokines, chemokines, MMPs, and toxic molecules (nitric oxide or free radicals) [33]. Efforts have been made to elucidate the mechanisms underlying the inflammatory responses to identify novel anti-inflammatory drug targets including the inflammation mediators such as TNF and IL-1 [34]. Several anti-TNF antibodies, including infliximab and adalimumab, have been approved by the FDA for the treatment of RA [20, 23]. However, long-term use of anti-TNF-*α* agents has also been reported to be closely associated with increasing risk of adverse events like serious infections, malignancies, skin, tuberculosis, and cancer [24]. Different from antibody-based agents, ebosin is a natural product that originates as secondary metabolites from *Streptomyces* sp.139 with efficacy and high safety properties, which may provide a more effective treatment option for RA.

Exopolysaccharides play a crucial role in several biological activities and have also remarkable industrial applications such as biothickeners in foods [35]. Numerous reports suggested that they can confer health benefits including anti-inflammation [36], cholesterol-lowering properties [37], antitumor activity [38], and antidiabetic activity [39]. EPSs isolated from *Trichoderma erinaceum* DG-312 exhibited a strong anti-inflammatory activity in inflamed mice [40]. Nowak et al. reported that EPS derived from *Lactobacillus rhamnosus* can significantly inhibit the production of arthritogenic antibodies, hence suppressing active CIA [41]. Recently, there has been growing interests in microbial EPS due to its broad medical applications in the field of immune regulation and antiviral activity, even against the coronavirus disease 2019 (COVID-19) [36, 42–45].

ERK1/2, JNK, and p38 MAPKs are the three major members of the MAPK family that respond to distinct signaling cascades [46]. The employment of p38 and JNK inhibitors has emerged as an attractive strategy to reduce both proinflammatory cytokine synthesis and its intracellular signaling [47]. Our results in this study showed that ebosin has a remarkably dose-dependent effect on reducing the phosphorylated p38, JNK1, and JNK2 MAPK protein levels in FLS induced by TNF-*α*. More than 50% phosphorylated protein was inhibited by ebosin at high dosage (400 mg/ml) that has no significant influence on the cell viability by cytotoxicity assay as described in our previous study [18]. Meanwhile, our research indicated that ebosin did not affect the expression of p42/44 MAPK (ERK1/2) which plays a key role in cell proliferation, differentiation, and migration [48].

The transcription factor NF-*κ*B, which initially exists in the cytoplasm in an inactive complex with I*κ*B, is a pivotal regulator in the regulation of inflammation and immune responses by eliciting the transcriptional responses following different stimuli such as TNF-*α* or IL-1*β* [28]. It has been reported that the activation of NF-*κ*B appears to precede disease onset, which suggested that inhibition of NF-*κ*B by different means may contribute to reduce the severity of disease [49]. In this study, we focused on the capacity of ebosin to counteract the level of NF-*κ*B in FLS induced by TNF-*α*. Our results showed decreased levels of phosphorylated IKK*α* and IKK*β* after treating with ebosin. Besides, ebosin was capable of downregulating the levels of phosphorylated I*κ*B*α*. EMSA indicated that ebosin decreased the DNA-binding activity of NF-*κ*B in the nucleus, probably through affecting the phosphorylation of NF-*κ*B itself. Furthermore, we also demonstrated that ebosin can significantly inhibit the NF-*κ*B nuclear translocation process using western blot analysis and fluorescence microscopy. All of the results mentioned above suggest that ebosin is an inhibitor of the NF-*κ*B-driven signaling pathway, which may be responsible for its anti-inflammatory effects *in vivo* [18].

MMP-1 and MMP-3, as targets of NF-*κ*B and major collagenolytic enzymes involved in tissue destruction, have been reported to be significantly elevated in the synovial fluid of RA patients [50]. In addition to MMPs, FLS produces chemokines into synovial tissue upon stimulation by proinflammatory cytokines that further enhance inflammation, hyperplasia, and cartilage destruction [51]. As a member of the CC subfamily of chemokines, RANTES is involved in the pathogenesis of RA by promoting leukocyte infiltration [52]. Another important chemokine, IL-8 has been reported to be strongly associated with leukocyte accumulation and inflammation in RA. In the current study, ELISA analysis demonstrated that MMP-1, MMP-3, RANTES, and IL-8 levels were suppressed by ebosin in FLS induced with TNF-*α*. These results suggested that inhibition of ebosin on NF-*κ*B activation contributes to reduce the secretion of MMPs and chemokines, thereby protecting RA patients from joint destruction.

In conclusion, this study has demonstrated that ebosin is capable of inhibiting the inflammatory responses induced by TNF-*α* in isolated FLS, acting as an effective *in vitro* inhibitor of the MAPKs and NF-*κ*B signaling pathways (Figure 6). It is assumed that the anti-inflammatory activity of ebosin is at least partially due to inhibition of these pathways. Therefore, ebosin may be developed as a potential candidate for the treatment of RA. Additional investigations to identify its clinical usefulness should be explored.

## Data Availability

The original data used to support the findings of this study are included within the article.

## Abbreviations

CIA: Collagen-induced arthritis
FLS: Fibroblast-like synoviocytes
MTT: 3-(4,5-Dimethylthiazol-2-yl)-2,5-diphenyltetrazolium bromide
IL-1*β*: Interleukin-1*β*
TNF-*α*: Tumor necrosis factor-*α*
MAPK: Mitogen-activated protein kinase
IKK: I*κ*B kinase
NF-*κ*B: Nuclear factor-kappa B
p38: p38 mitogen-activated protein kinase
JNK: Jun N-terminal kinase
ELISA: Enzyme-linked immunosorbent assay
EMSA: Electrophoretic mobility shift assay
MMP: Matrix metalloproteinase
RANTES: Reduced upon activation normal T expression and secreted
IL-8: Interleukin-8
TRAF2: TNF receptor-associated factor2
RIP: Receptor interacting protein
DMEM: Dulbecco's modified Eagle's medium
DMSO: Dimethyl sulfoxide
RIPA: Radio immunoprecipitation assay
HRP: Horseradish peroxidase
PVDF: Polyvinylidene fluoride
EDTA: Ethylene diamino tetraacetic acid
PBS: Phosphate-buffered saline
BCA: Bicinchoninic acid.
